# Sirtuin 6 activation rescues the age-related decline in DNA damage repair in primary human chondrocytes

**DOI:** 10.18632/aging.205394

**Published:** 2023-12-09

**Authors:** Michaela E. Copp, Jacqueline Shine, Hannon L. Brown, Kirti R. Nimmala, Oliver B. Hansen, Susan Chubinskaya, John A. Collins, Richard F. Loeser, Brian O. Diekman

**Affiliations:** 1 Thurston Arthritis Research Center, University of North Carolina at Chapel Hill, Chapel Hill, NC 27599, USA; 2 Joint Department of Biomedical Engineering, University of North Carolina and North Carolina State University, Raleigh, NC 27606, USA; 3 School of Medicine, University of North Carolina at Chapel Hill, Chapel Hill, NC 27599, USA; 4 Department of Pediatrics, Rush University Medical Center, Chicago, IL 60612, USA; 5 Department of Orthopedic Surgery, Thomas Jefferson University, Philadelphia, PA 19144, USA; 6 Division of Rheumatology, Allergy, and Immunology, University of North Carolina at Chapel Hill, Chapel Hill, NC 27599, USA

**Keywords:** SIRT6, MDL-800, cartilage, aging, comet assay

## Abstract

While advanced age is widely recognized as the greatest risk factor for osteoarthritis (OA), the biological mechanisms behind this connection remain unclear. Previous work has demonstrated that chondrocytes from older cadaveric donors have elevated levels of DNA damage as compared to chondrocytes from younger donors. The purpose of this study was to determine whether a decline in DNA repair efficiency is one explanation for the accumulation of DNA damage with age, and to quantify the improvement in repair with activation of Sirtuin 6 (SIRT6). After acute damage with irradiation, DNA repair was shown to be more efficient in chondrocytes from young (≤45 years old) as compared to middle-aged (50–65 years old) or older (>70 years old) cadaveric donors. Activation of SIRT6 with MDL-800 improved the repair efficiency, while inhibition with EX-527 reduced the rate of repair and increased the percentage of cells that retain high levels of damage. In addition to affecting repair after acute damage, treating chondrocytes from older donors with MDL-800 for 48 hours significantly reduced the amount of baseline DNA damage. Chondrocytes isolated from the knees of mice between 4 months and 22 months of age revealed both an increase in DNA damage with aging, and a decrease in DNA damage following MDL-800 treatment. Lastly, treating murine cartilage explants with MDL-800 lowered the percentage of chondrocytes with high p16 promoter activity, which supports the concept that using SIRT6 activation to maintain low levels of DNA damage may prevent the initiation of senescence.

## INTRODUCTION

Osteoarthritis (OA) is a chronic joint disease affecting approximately 13% of the US adult population and is characterized by the degradation of articular cartilage, synovial inflammation, and subchondral bone remodeling [1–3]. As no disease-modifying therapies for OA have been FDA approved to date [4], the main options available to OA patients are pain management and eventual total joint replacement, leading to extensive societal and economic burdens [5]. While a number of risk factors have been associated with OA – obesity, biological sex, joint injury, and genetics – the leading risk factor is older age [6]. While progress continues to be made, the biological mechanisms linking aging and OA prevalence remain largely unknown [7].

Hypo-replicative cell types such as neurons, hematopoietic stem cells, and chondrocytes tend to accumulate sites of persistent DNA damage during aging, due at least in part to the lack of access to repair mechanisms that are only present in S phase [8–10]. As measured by the alkaline comet assay [11, 12], we showed that chondrocytes isolated from older cadaveric donors, despite no known clinical history of OA or severe macroscopic cartilage damage, harbor high levels of DNA damage [13]. The increased DNA damage is therefore present at a time when cartilage is susceptible to degradation (older age), but before significant OA progression has occurred. One objective of this study was to determine whether a reduced efficiency of DNA damage repair with aging is one potential cause of DNA damage accumulation.

Sirtuin 6 (SIRT6) is a nuclear-localized NAD (+)-dependent deacetylase that has been shown to play numerous important roles in cellular processes that become dysregulated with aging [14–17]. SIRT6 quickly localizes to sites of DNA damage and initiates chromatin remodeling to facilitate the recruitment and activity of proteins involved in DNA repair [18–22]. Prior work has indicated that SIRT6 is a critical factor in joint tissue homeostasis [23–26]. Our team has shown decreased SIRT6 activity in chondrocytes with aging, despite similar gene expression and protein values [26]. Another group showed a reduction in protein level of SIRT6 in OA samples collected from joint replacement surgery as compared to normal cartilage collected from amputation surgery [25]. Small molecules can be used to either increase or decrease the deacetylase activity of SIRT6. MDL-800 is an allosteric activator that increased activity by up to 22-fold in a peptide-based assay [27], and decreased H3K9ac (H3K9 is a known target of SIRT6 deacetylase activity) in primary human chondrocytes at 12.5 μM [28]. In contrast, EX-527 is an inhibitor that stabilizes the closed conformation of sirtuins (including but not limited to SIRT6) [29] and blocks 67% of recombinant SIRT6 activity within 15 minutes [26]. The second objective of this study was to examine how modulating SIRT6 activity impacts the repair of DNA.

Prior work completed in our lab has demonstrated that primary human chondrocytes accumulate damage in a linear manner with age, predominantly driven by strand breaks to the DNA [13]. The third objective of this study was to determine the extent to which MDL-800 can reduce the high levels of DNA damage present in chondrocytes from older donors. Similarly, we investigated whether murine chondrocytes show increased DNA damage with age and whether MDL-800 treatment is sufficient to reverse damage in this important model species.

Persistent DNA damage is a common feature in numerous contexts that drive cellular senescence and other age-related dysfunction [30]. A causative role for DNA damage in senescence is supported by studies that apply exogenous DNA damage or disrupt DNA repair pathways; however, the inverse has been more challenging to test experimentally – does enhanced DNA repair efficiency mitigate senescence [31]? To provide an initial assessment of this possibility, a fourth goal was to treat murine hip cartilage explants with MDL-800 and assess senescence burden using a p16^tdTom^ allele [32].

In this study, we use irradiation as an acute model of DNA damage to bring the level of damage to equivalent levels across chondrocytes from donors of various ages. We show that the DNA repair efficiency of chondrocytes deteriorates throughout life but can be enhanced by activating SIRT6. Further, we demonstrate that SIRT6 activation is sufficient to reduce the accumulated DNA damage that arises with aging in human and murine chondrocytes. These results establish SIRT6 activation as one approach to improve DNA damage repair in chondrocytes, which could potentially mitigate the age-related decline in chondrocyte function.

## RESULTS

### Decreased DNA damage repair efficiency with aging in primary human chondrocytes

**Figure 1:**
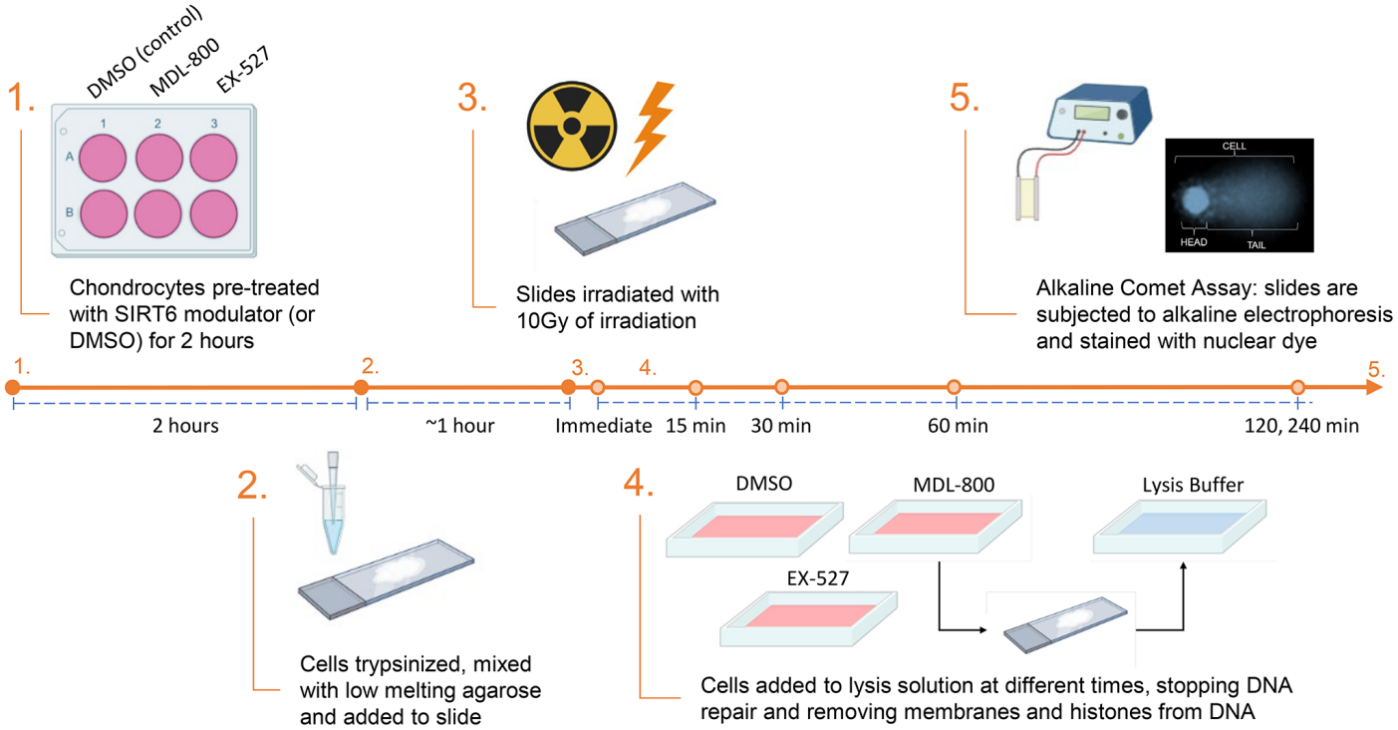
**Experimental design for the results shown in Figures 3 and 4.** For the data in Figures 2 and 3 there was no pre-treatment and steps 2–5 were completed as shown (with standard cell culture media used for the repair phase).

**Figure 2:**
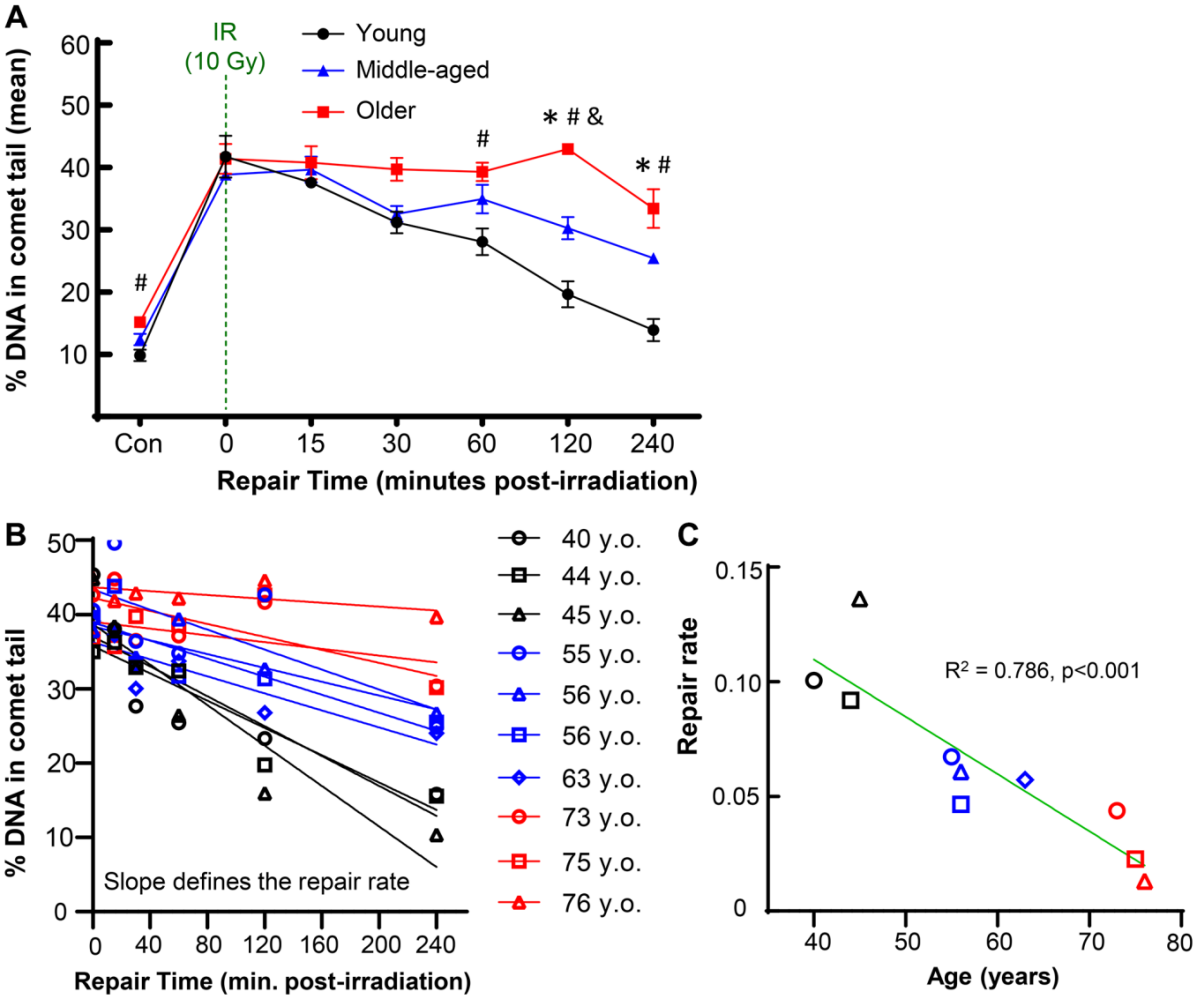
**Effect of donor age on repair after acute DNA damage.** Primary human chondrocytes from young (*n* = 3, ≤45 years), middle-aged (*n* = 4, 50–65 years), and older (*n* = 3, >70 years) donors were prepared in gels on microscope slides, irradiated with 10 Gy (or not for control), and allowed to repair for various amounts of time. (**A**) The percentage of DNA in comet tails for all cells was averaged for each donor, and the mean of all donors per age group is shown (mean ± SEM). Repair time, age, and their interaction were significant sources of variation (2-way repeated measures ANOVA). Significant differences between groups at each time point (Tukey’s multiple comparisons test, *p* < 0.05) are denoted by symbols: (^*^) = young vs. middle, (^#^) = young vs. old, (^&^) = middle vs. old. (**B**) The repair rate of each donor was calculated by plotting the % DNA in comet tail against repair time. The slope of the linear regression was used to define the repair rate for each donor. (**C**) The repair rate is plotted against age and the slope of the linear regression was significantly non-zero (*p* = 0.0006).

To investigate how aging impacts the repair capacity of chondrocytes, we used irradiation to apply an acute bolus of damage to cells and monitored DNA damage by the comet assay at time points of 15, 30, 45, 60, 120, and 240 minutes after damage. This irradiation model allowed us to apply nearly instantaneous damage to the cells and conduct a precise time-course study of repair by transferring the slides directly to the lysis buffer (experimental approach in Figure 1). Importantly, chondrocytes from distinct age ranges of young (≤45 years old), middle (50–65 years old), and older (>70 years old) adults had a similar amount of DNA damage immediately after irradiation, indicating that this bolus of damage was sufficient to overcome the background differences in accumulated damage. The ability of the chondrocytes to resolve DNA damage from this equal starting point over the course of 4 hours was impaired in the middle-aged and older donors as compared to the young donors (Figure 2A). The older donors had a significantly higher percentage of DNA in comet tails as compared to the middle and/or younger donors at 60, 120, and 240 minutes (*p* < 0.05, multiple comparisons test). Representative images for donors of each age group across the full experimental time course are provided in Supplementary Figure 1. By 4 hours post-irradiation, most of the damage was resolved in chondrocytes from younger donors, whereas the average percentage of DNA in comet tails remained elevated for both middle-aged and older donors. The repair rate was calculated for each donor by determining the slope of the linear regression when the percentage of DNA in the comet tail is plotted against the time course of repair (Figure 2B). When the repair rate is then plotted against the age of each individual donor, there is a strong inverse relationship between the efficiency of repair and donor age (Figure 2C, *p* < 0.001, slope significantly non-zero, R^2^ = 0.786).

**Figure 3:**
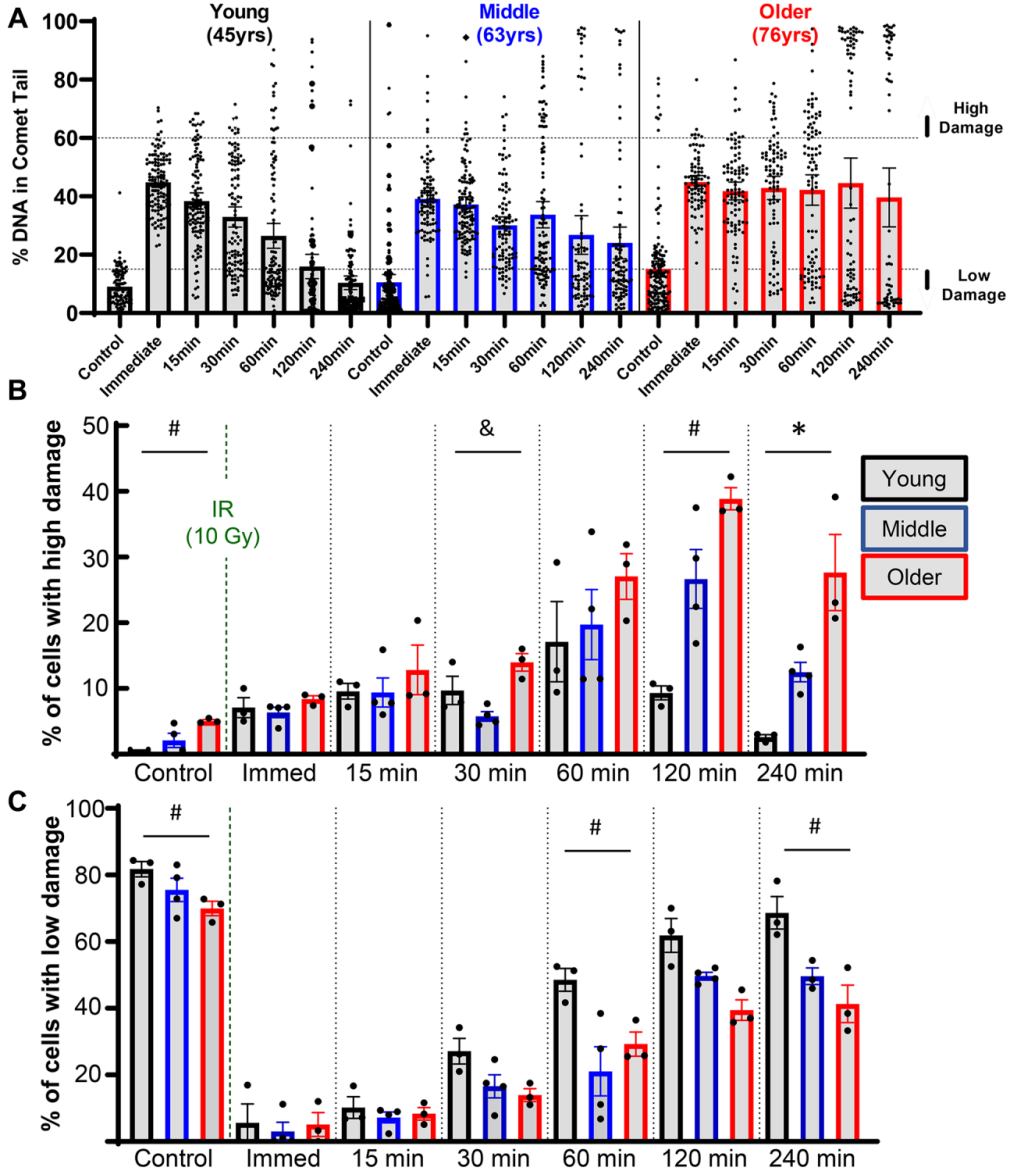
**Effect of donor age on success of resolving acute DNA damage.** (**A**) Plots showing all individual cells of representative young, middle, and older donors. (**B**) The percentage of cells with high levels of DNA damage (>60% of DNA in comet tails) for each donor, with bars showing the mean ± SEM of all donors per age group. (**C**) Same as B except now showing the percentage of cells with low levels of DNA damage (<15% of DNA in comet tail). For B and C the repair time, age, and their interaction were significant sources of variation by 2-way repeated measures ANOVA. Significant differences between groups at each time point (Tukey’s multiple comparisons test, *p* < 0.05) are denoted by symbols: (^*^) = young vs. middle, (^#^) = young vs. older, (^&^) = middle vs. older.

Insight can be gained by assessing the distribution of damage within individual cells for each donor, as shown for representative young, middle, and older donors (Figure 3A). Of note, there was a bifurcated response in the older donors, with a significant fraction of cells retaining very high levels of damage (above the dotted line that demarcates 60% of the DNA in comet tails). When quantified across all donors, 27.6% of chondrocytes in the older group retained this high level of damage at 4 hours, whereas this percentage was 12.5% and 2.6% for middle-aged and younger donors, respectively (Figure 3B). Analysis of chondrocytes with <15% DNA in comet tails at 4 hours showed that 68.7%, 49.6%, and 41.3% of cells from young, middle-aged, and older donors, respectively, repaired the damage from irradiation to near-baseline levels (Figure 3C).

### SIRT6 activation and inhibition affects the repair efficiency of chondrocytes

**Figure 4:**
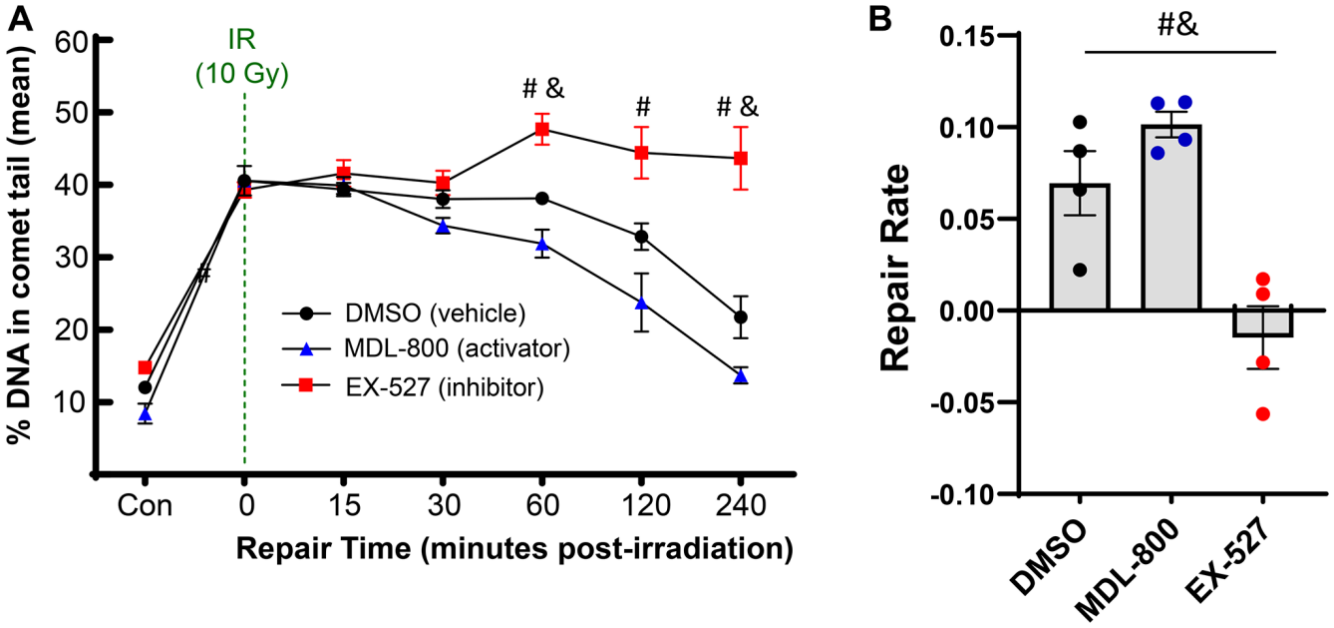
**Effect of SIRT6 activation and inhibition on chondrocyte repair rate.** Chondrocytes from middle-aged donors were pre-treated with 20 μM MDL-800, 10 μM EX-527, or vehicle (DMSO) for 2 hours before trypsinization, gel encapsulation, and irradiation. Treatment continued during the repair phase. (**A**) The percentage of DNA in comet tails for all cells were averaged for each donor, and the mean of all donors per condition is shown (mean + SEM). Repair time, treatment, and their interaction were significant sources of variation (2-way repeated measures ANOVA). Significant differences between groups at each time point (Tukey’s multiple comparisons test, *p* < 0.05) are denoted by symbols: (^*^) = DMSO vs. MDL, (^#^) = MDL vs. EX, (^&^) = DMSO vs. EX). (**B**) The repair rate of chondrocytes is improved by MDL-800 treatment and inhibited by EX-527 treatment. Statistics as in A (repair rate calculated by calculating linear regression of percent DNA in comet head over 240 minutes; mean + SEM).

As SIRT6 has been shown to coordinate DNA repair in other cell types, we sought to study how modulating SIRT6 activity impacts the efficiency of DNA repair in chondrocytes. Using the same irradiation and comet assay system, chondrocytes from middle-aged donors were pre-treated for 2 hours with MDL-800 (SIRT6-specific activator), EX-527 (inhibitor of SIRT6 and SIRT1), or DMSO (vehicle control). Following encapsulation in low-melt agarose and irradiation, the slides were placed back into media baths with their respective treatments for recovery, such that the cells were receiving SIRT6 activation/inhibition for the entirety of the repair phase (experimental approach in Figure 1). When assessed by repeated measures two-way ANOVA without consideration of EX-527 treatment, MDL-800 treated groups showed lower DNA damage as compared to DMSO in middle-aged donors (Figure 4A, main effects *p*-value = 0.005). Similarly, when DMSO and MDL-800 were compared in chondrocytes from older donors (>70 years), there was reduced damage with MDL-800 treatment at 30, 60, 120, and 240 minutes (Supplementary Figure 2A). Further, MDL-800 reduced the percentage of cells with high damage (>60% DNA in comet tails) at 4 hours from 20.1% to 4.9% (Supplementary Figure 2B). When EX-527 was also considered in the ANOVA for middle-aged donors, this inhibitor showed strong effects with significantly more DNA damage as compared to the DMSO and/or MDL-800 groups at baseline, 60, 120, and 240 minutes of repair (Figure 4A). Comparing the repair rate of the different treatment conditions showed a significant decrease in the SIRT6 inhibited group compared to the DMSO and MDL-800 treated groups (Figure 4B).

**Figure 5:**
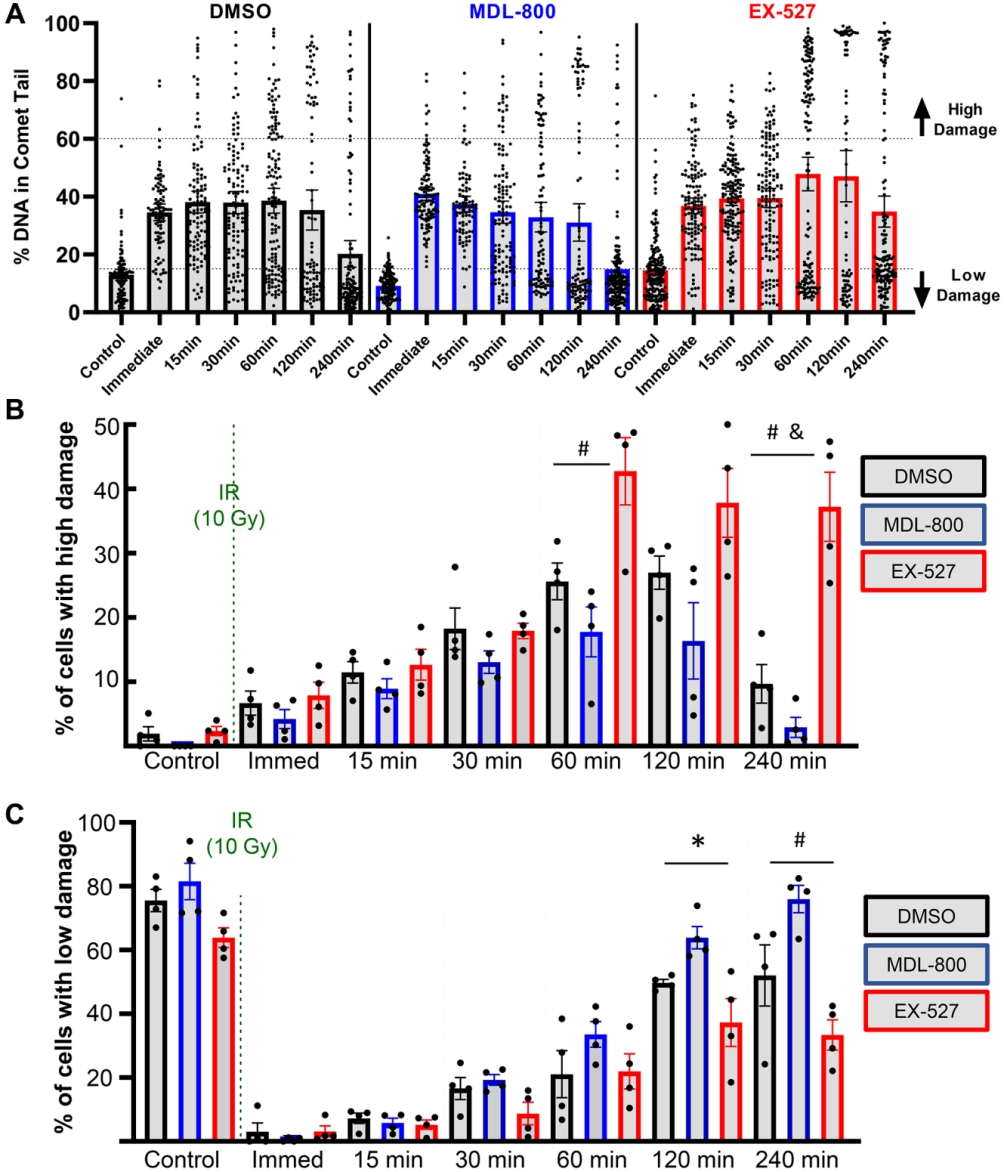
**Effect of SIRT6 modulation on chondrocyte repair efficiency.** (**A**) Plots show all individual cells of a representative donor treated with DMSO, MDL-800, or EX-527. (**B**) The percentage of cells with high levels of DNA damage (>60% of DNA in comet tails). (**C**) The percentage of cells with low levels of DNA damage (<15% of DNA in comet tails) following DMSO, MDL-800, or EX-527 treatment. Significant differences between groups at each time point (Tukey’s multiple comparisons test, *p* < 0.05) are denoted by symbols: (^*^) = DMSO vs. MDL, (^#^) = MDL vs. EX, (^&^) = DMSO vs. EX).

The all-cell plot shows a striking increase in the percentage of individual cells that retain high levels of DNA damage in the EX-527 group (Figure 5A). At 4 hours, 37.2% of cells in the EX-527 group still had greater than 60% of the DNA in comet tails, while only 2.9% of cells in the MDL-800 group had high levels of damage (Figure 5B). When comparing the percentage of cells with low levels of DNA damage, MDL-800 treatment significantly increased the likelihood that cells can restore near-baseline levels of damage at two and four hours post-irradiation (Figure 5C).

We assessed whether damage in the form of 10 Gy irradiation caused extensive apoptosis and whether this was altered by SIRT6 modulation. We first used flow cytometry to assess cell death (near-IR dye that enters dead cells) as well as apoptosis (Caspase 3/7) at four hours after IR. Across three donors, all conditions showed greater than 85% viability and the majority of the dead cells were also positive for Caspase 3/7 (Supplementary Figure 3A, 3B). To confirm viability within low-melt agarose gels, Calcein AM was used to mark live cells and Ethidium homodimer to mark dead cells. By assessment of fluorescent images four hours after IR, again more than 85% viability was observed across all conditions (Supplementary Figure 3C, 3D).

### SIRT6 activation decreases DNA damage associated with older age

**Figure 6:**
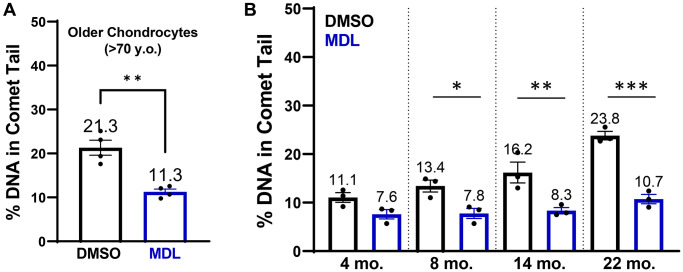
**SIRT6 activation in chondrocytes from older human donors and mice.** (**A**) Chondrocytes derived from cadaveric ankle cartilage of older donors (>70 years) were treated with 20 μM MDL-800 or vehicle (DMSO) for 48 hours. Stats by paired *t*-test. (**B**) Murine chondrocytes were isolated and treated for 48 hours with DMSO or 20 μM MDL-800. Analysis by two-way ANOVA showed significant effects of age, treatment, and their interaction. Asterisks denote significant treatment effects by Sidak’s multiple comparisons test, with ^*^p < 0.05, ^**^p < 0.01, ^***^p < 0.001.

Having shown that SIRT6 activity affects the repair capacity of chondrocytes in response to an acute bolus of damage, we wanted to test whether MDL-800 could also repair long-standing naturally accumulated damage. In previous studies, we have established that there is higher DNA damage in chondrocytes from older donors, with a linear regression showing that donors at age 40 have ~10% DNA in comet tails and donors at age 75 have ~27% [13]. Here, we treated chondrocytes isolated from older cadaveric donors for 48 hours with either 20 μM MDL-800 or vehicle control (DMSO). The MDL-800 treated chondrocytes showed significantly lower levels of DNA damage (mean: 11.3% of DNA in comet tails) as compared to the DMSO groups (21.3%) (Figure 6A, *p* = 0.0031, paired *t*-test).

### MDL-800 treatment reduces DNA damage in aged murine chondrocytes

Mice are a commonly used model species for investigations of mammalian aging and thus we sought to determine whether MDL-800 can also lower the DNA damage that accumulates with age in murine chondrocytes. Chondrocytes from the knee were isolated and then treated with MDL-800 (20 μM) or DMSO control for 48 hours in monolayer before comet analysis. DNA damage increased in the DMSO-treated groups with age, with the percentage of DNA in comet tails approximately doubling from 4 to 22 months of age (Figure 6B). MDL treatment consistently lowered DNA damage in all age groups, with significant reductions at 8, 14, and 22 months of age (Figure 6B, *p* < 0.05, multiple comparisons test).

### MDL-800 treatment during culture limits senescence induction in murine cartilage explant model

**Figure 7:**
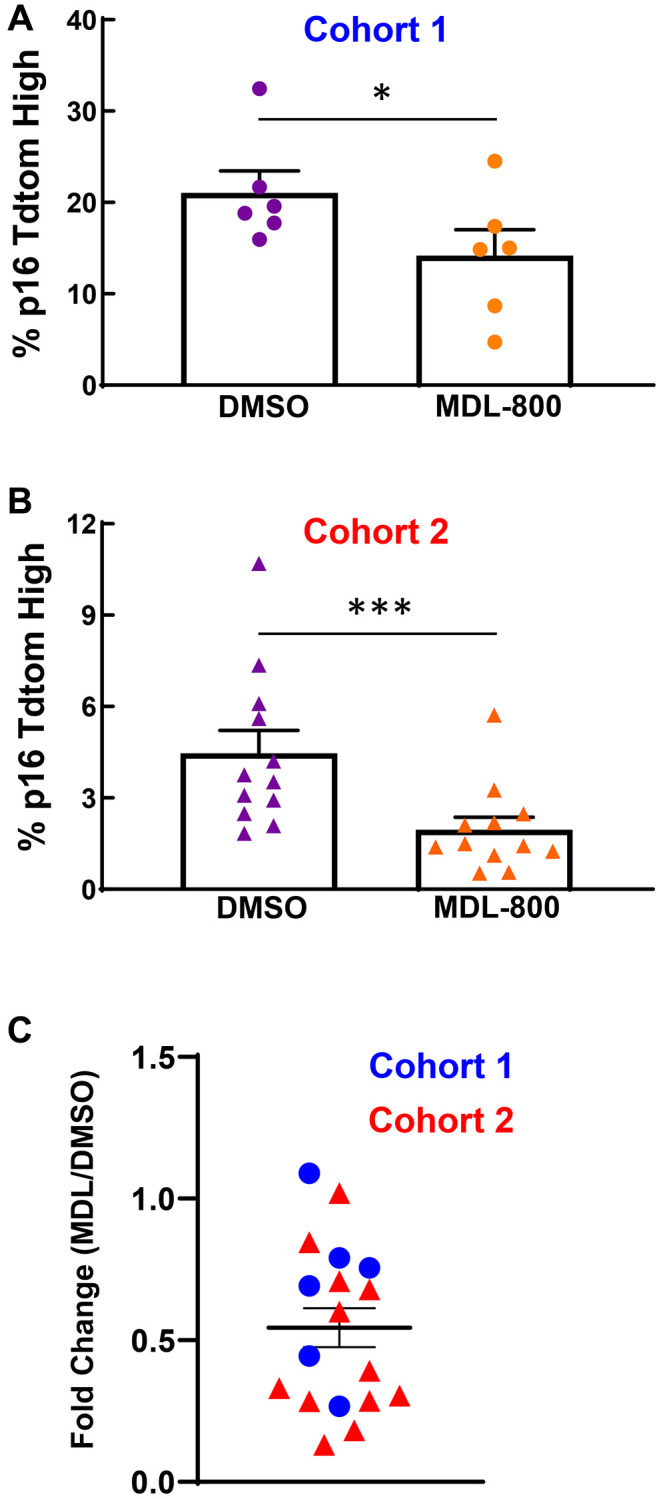
**SIRT6 activation limits senescence burden in murine cartilage explants.** (**A**) Femoral cap cartilage was obtained from each hindlimb of 3-week-old p16^tdTom^ mice and cultured for three weeks under senescence inducing conditions with either 20 μM MDL-800 or DMSO control. Analysis of the percentage of cells positive for tdTomato performed by flow cytometry. Data from Cohort 1 were normally distributed by Shapiro-Wilk and thus paired *t*-test was applied. (**B**) Same as panel A but a different cohort of mice. Data from cohort 2 were not normally distributed by Shapiro-Wilk and thus Wilcoxon matched-pairs signed rank test was applied. (**C**) For each mouse (blue circles: cohort 1; red triangles: cohort 2), the percentage of tdTomato-positive cells in the MDL explant was normalized to the DMSO explant. Asterisks denote statistical significance, with ^*^p < 0.05, ^***^p = 0.001.

We previously established a model that uses transforming growth factor beta and fibroblastic growth factor to initiate senescence within cartilage explants harvested from young p16^tdTom^ mice [32]. Assessment of the tdTomato fluorescence signal by flow cytometry provides a quantitative readout of p16 promoter activity, which is an established biomarker of senescence [33]. We utilized this model system to determine whether continuous SIRT6 activation during the three-week culture period would limit the initiation of senescence. In a first cohort, 20 μM MDL-800 treatment decreased the percentage of p16^tdTom^-high cells as compared to the DMSO control (Figure 7A, 21.0% vs. 14.2%, *p* < 0.05, paired *t*-test). A second cohort showed similar results but had a lower overall percentage of p16^tdTom^-high cells (Figure 7B, 4.5% vs. 2.0%, *p* = 0.001, Wilcoxon paired test). Across both cohorts, the ratio of senescence for the MDL-800 explant to DMSO explant of each mouse demonstrated that MDL-800 treatment halved the percentage of senescent cells (Figure 7C, ratio = 0.54, *p* < 0.01, one-sample Wilcoxon).

## DISCUSSION

In the present study, we found that (1) advanced age negatively impacts the ability of chondrocytes to repair DNA damage, (2) modulating SIRT6 activity affects the repair capacity of chondrocytes, (3) activating SIRT6 with MDL-800 can aid in repairing DNA damage that had accumulated during physiological aging, and (4) treatment with MDL-800 can mitigate the induction of senescence in murine cartilage explants. The first two findings made use of irradiation to initiate a bolus of damage. This system was particularly valuable in that the level of damage immediately following irradiation was consistent across all ages and treatment groups, allowing us to directly compare the progressive reduction in DNA damage over time.

There is growing evidence that the efficiency of DNA damage repair declines with age (reviewed in [34] and [35]). Previous work has largely been performed in fibroblasts and lymphocytes, but the current study confirms that aging also affects the repair of DNA damage in primary human chondrocytes. We used the alkaline comet assay to provide a sensitive and quantitative measure of DNA damage levels. Upon placement in a lysis solution, strand breaks and other forms of damage (i.e., abasic sites) relax the supercoiled DNA loops of the nucleus, enabling easier movement of the DNA through the agarose gel when an electric field is applied [11, 12]. As a result, damaged DNA produces a “comet tail” while intact DNA remains in the “comet head”. In this study, we noted a substantial and mostly linear decrease in the repair rate of chondrocytes with age. This finding may partially explain the linear increase in accumulated DNA damage with aging that we have previously demonstrated [13]. One advantage of this assay is the single-cell nature of the readout. This allowed us to observe that chondrocytes from older donors had a larger percentage of cells that showed very little repair and instead retained a high damage burden at four hours post-irradiation. This finding aligns with a previous study in lymphocytes that showed the primary difference with age in response to irradiation was the increased subset of cells that retained high damage [36].

SIRT6 is involved in numerous biological processes with relevance to aging [37], including a role in multiple DNA damage repair pathways [18, 19, 38–40]. Given the selectivity of MDL-800 for SIRT6 [27], we were able to show that activation of SIRT6 is sufficient to repair approximately half of the accumulated damage in chondrocytes from older human donors and from older mice. For human chondrocytes, 48 hours of treatment with MDL-800 lowered the percentage of DNA in comet tails from 21.3% to 11.3%. Based on the linear regression calculated from 25 donors ranging in age from 34 to 78 years old in Copp et al. [13], MDL-800 treatment was therefore able to eliminate the equivalent of ~34 years worth of damage.

Cellular senescence is a phenotypic state characterized by stable cell cycle arrest in response to intrinsic or extrinsic stress [41]. The accumulation of senescent cells has been associated with numerous aging-related diseases and likely plays a role in OA pathogenesis [42, 43]. However, less is known regarding the biological processes and environmental cues that prime chondrocytes to become senescent. Evidence supports the notion that DNA damage is a causative factor that drives senescence and other features of aging [30, 31], and other studies have linked DNA damage with chondrocyte dysfunction during OA [44]. Our previous work also supports a causal role for DNA damage in chondrocyte senescence, as the application of 10 Gy irradiation (which recapitulates the level of DNA damage in older donors [13]) is capable of inducing senescence in cartilage explants when paired with a mitogenic stimulus [45]. In this study, we used our established murine hip cartilage explant system for senescence induction in p16^tdTom^ mice [32]. When MDL-800 was provided over as a three-week culture period, the senescence burden was approximately half that seen in matched explants that were treated with a vehicle control.

Other studies have provided *in vivo* and mechanistic support for the importance of Sirt6 in maintaining chondrocyte function. Collins et al. demonstrated that cartilage-specific deletion of Sirt6 via Aggrecan-CreERT2 and tamoxifen at 12 weeks resulted in greater post-traumatic and age-related OA [28]. Sirt6 deficient cartilage had reduced activity of the insulin-like growth factor/Akt pathway, and adenovirus overexpression or activation of SIRT6 by MDL-800 enhanced this anabolic signaling in human chondrocytes [28]. Another study used the Col2-CreERT2 and tamoxifen at 8 weeks to show that Sirt6 deficiency exaggerated chondrocyte senescence and OA, with increased inflammatory signaling through IL-15/JAK3/STAT5 [25]. Further, intra-articular injection of adenovirus-Sirt6 or the introduction of nanoparticles releasing MDL-800 mitigated OA caused by destabilization of the medial meniscus surgery [25]. When paired with the results of the current study, these data suggest that SIRT6 activation may prevent senescence and OA through multiple mechanisms that promote cartilage health.

There are important limitations to this study. One is that the alkaline comet assay detects single-strand breaks (SSBs), double-strand breaks (DSBs) and alkali-labile forms of base damage such as apurinic/apyrimidinic sites [46]. Our previous study using a “two-tailed” comet assay that employs a buffer pH change between two electrophoresis runs showed that 10 Gy IR initiates both strand breaks and base damage in primary chondrocytes [13]. Given that the ratio of SSBs: DSBs is estimated at ~20:1 in the acute phase after IR [47], the contribution of DSBs to the comet results up to 4 hours is predicted to be minimal. Therefore, the differential repair due to donor age and SIRT6 modulation at time points of 1, 2, and 4 hours should be interpretated as representing alterations in the efficiency of repair for direct SSBs, base damage, and base damage intermediates [48]. Another limitation is that EX-527 inhibits SIRT1 as well as SIRT6 and thus the observed reduction in DNA repair efficiency may not be entirely due to SIRT6, especially given the possibility of cooperation between these two sirtuins [49]. While the high selectivity of MDL-800 gives confidence that the accelerated repair is due to SIRT6 activation, further work using RNA interference, genome editing, or more selective inhibitors would be required to fully parse the effects of SIRT1 and SIRT6. A final limitation is that irradiation causes multiple forms of DNA damage, including complex lesions that are particularly challenging to repair efficiently [50]. Future work with agents that initiate specific types of DNA would be able to parse the repair pathways that are most affected by age and those most amenable to enhancement with SIRT6 activation.

In conclusion, the findings presented here support the hypothesis that the efficiency of DNA damage repair declines with age in chondrocytes and that SIRT6 activation improves repair both in response to an acute irradiation challenge and in the context of age-related damage accumulation. These results emphasize the critical role of SIRT6 in DNA repair and support further studies investigating the use of MDL-800 (or alternative SIRT6 activators) in mitigating senescence induction and ameliorating OA development.

## MATERIALS AND METHODS

### Isolation and culture of primary human chondrocytes

Primary human chondrocytes were isolated from the ankle cartilage of cadaveric donors without a history of OA and with grades between 0 and 2 on the modified Collins grade [51]. Use of this tissue source was approved by the Institutional Review Boards of Rush University and the University of North Carolina at Chapel Hill; patient consent is not applicable due to the use of cadaveric tissue. For the study presented in Figures 2, 3, ages of donors were in three groups: younger (40, 44, 45 years old); middle-aged (56, 56, 63 years old); and older (73, 75, 76 years old). For the study presented in Figures 4, 5, the donors used were middle-aged (51, 54, 54, 55, 56, 56, 60, 63). For the study presented in Figure 6A, the ages of the donors were 74, 75, 75, and 76. To isolate the primary chondrocytes, full-thickness cartilage shards were digested with 2 mg/ml Pronase (1 hour) followed by overnight incubation with 3.6 mg/ml Collagenase *P* at 37^o^C in 5% serum media [52]. The isolated chondrocytes were plated at a concentration of ~1 × 10^5^ cells per cm^2^ in DMEM/F12 supplemented with 10% FBS, penicillin and streptomycin, gentamicin, and amphotericin B to recover from isolation and frozen in Recovery^™^ Cell Culture Freezing Medium. Chondrocytes were thawed and plated for ~2–3 days of passage 1 culture before harvest and resuspension in comet gels for irradiation.

### Isolation of primary murine chondrocytes

The cartilage surfaces of the femurs and tibiae of C57BL/6 mice were dissected for chondrocyte isolation. Chondrocytes were isolated via Pronase (2 mg/ml, 1 hour in serum-free media) and collagenase *P* (500 μg/ml, overnight in 10% serum media) from mice aged 4, 8, 14, and 22 months of age (*n* = 3 each). Chondrocytes were cultured for ~3 days to recover before treatment.

### SIRT6 activation and inhibition treatment

The small molecule MDL-800 (Sigma) was used at a concentration of 20 μM to activate SIRT6. Conversely, EX-527 (Selleck) was used at a concentration of 10 μM to inhibit SIRT6 activity. When testing the effect of SIRT6 modulation on DNA repair following acute damage (Figures 4, 5), primary chondrocytes were pre-treated with either DMSO (vehicle control, concentration matching the DMSO used with MDL-800), MDL-800, or EX-527 for 2 hours prior to harvest for irradiation experiments. For experiments testing the reduction of accumulated DNA damage in chondrocytes from older cadaveric donors and mice, cells were treated with DMSO or 20 μM MDL-800 for 48 hours.

### Acute irradiation repair model and comet assay protocol

A schematic depicting the irradiation repair model is shown in Figure 1. After trypsinization, chondrocytes were prepared for the comet assay as described [13], with adjustments made to measure DNA damage levels at specific time points following irradiation. Briefly, cells were mixed 1:10 with 1% low melting agarose and coated onto a Superfrost slide. The slides were placed in a media bath and irradiated with 10 Gy X-ray (RS2000 Biological Irradiator), with one slide not irradiated as a control group. The slides were moved to an incubator with their appropriate media for various amounts of time for recovery and then added to a lysis solution at the indicated time point – immediate (no recovery after IR), 15 min., 30 min., 60 min., 120 min., and 240 min. The lysis solution was prepared by mixing 2.5 M NaCl, 0.1 M disodium EDTA, 10 mM Tris base, 0.2 M NaOH, 0.1% sodium lauryl sarcosinate, and 1% Triton X-1000, and adjusting the solution to a pH of 10. After overnight incubation in the lysis solution at 4°C, the slides were added to an alkaline electrophoresis solution (200 mM NaOH, 1 mM disodium EDTA, pH >13) for 30 minutes. Next, the slides were placed into an electrophoresis chamber and an electric field of 1 V/cm for 20 minutes was applied. Slides were washed with dH_2_O and stained with NucBlue^™^ (R37605; Thermo Fisher Scientific). Fluorescence images were captured with an EVOS M5000 microscope (AMF5000; Thermo Fisher Scientific). Image analysis and comet quantification were performed for approximately 100 cells per condition using the Open Comet plugin software in ImageJ.

### Senescence induction in murine hip cartilage explants

Cartilage from approximately three-week-old p16^tdTom^ mice was isolated and cultured as previously described [32]. Briefly, femoral cap cartilage explants were cultured with 1 ng/ml transforming growth factor beta and 5 ng/ml basic fibroblastic growth factor along with 10% serum media for three weeks. Matched explants from each mouse were treated with 20 ng/ml MDL-800 or an equivalent amount of DMSO at each feed. Tissue was digested with collagenase and directly analyzed for the percentage of cells with tdTomato fluorescence using an Attune NxT Flow Cytometer (Thermo Fisher Scientific).

### Apoptosis assessment

Chondrocytes in monolayer were pre-treated for 2 hours with MDL-800 or EX-527, irradiated with 10 Gy, and cultured a further 4 hours in treatment media. Cells were then trypsinized and assessed for viability and apoptosis by flow cytometry using a near-IR fixable live/dead dye (Thermo Fisher Scientific L34975) and Caspase 3/7 (Thermo Fisher Scientific C10430). For assessment of viability within low melt agarose gels after IR, cells were pre-treated for 2 hours before resuspension, irradiation, and 4 hours of additional culture in treatment media. The cell-laden gels were then stained with Calcein AM (Thermo Fisher Scientific C3099) to mark live cells and Ethidium homodimer (Thermo Fisher Scientific E1169) to mark dead cells. Images were taken on an EVOS m5000 (Thermo Fisher Scientific) and quantification performed by manual counting.

### Statistical analysis

Comet data were analyzed and plotted using GraphPad Prism 9. Statistical analysis was performed using paired *t*-test, Wilcoxon matched-pairs signed rank test (if data were not normally distributed as assessed by Shapiro-Wil test), two-way ANOVA, or two-way repeated measures ANOVA. Outliers were removed based on ROUT with *Q* = 1%. Multiple comparison test used either Sidak’s (two treatment groups) or Tukey’s (three treatment groups) within each time point.

## Supplementary Materials

Supplementary Figures
