# Supplementary material for: Sirtuin 6 activation rescues the age-related decline in DNA damage repair in primary human chondrocytes
Source: Aging (Albany NY). 2023 Dec 9;15(23):13628–45. doi: 10.18632/aging.205394 (PMC10756124; doi:10.18632/aging.205394)
Supplement: Supplementary Figures [file aging-15-205394-s001.pdf]

SUPPLEMENTARY FIGURES

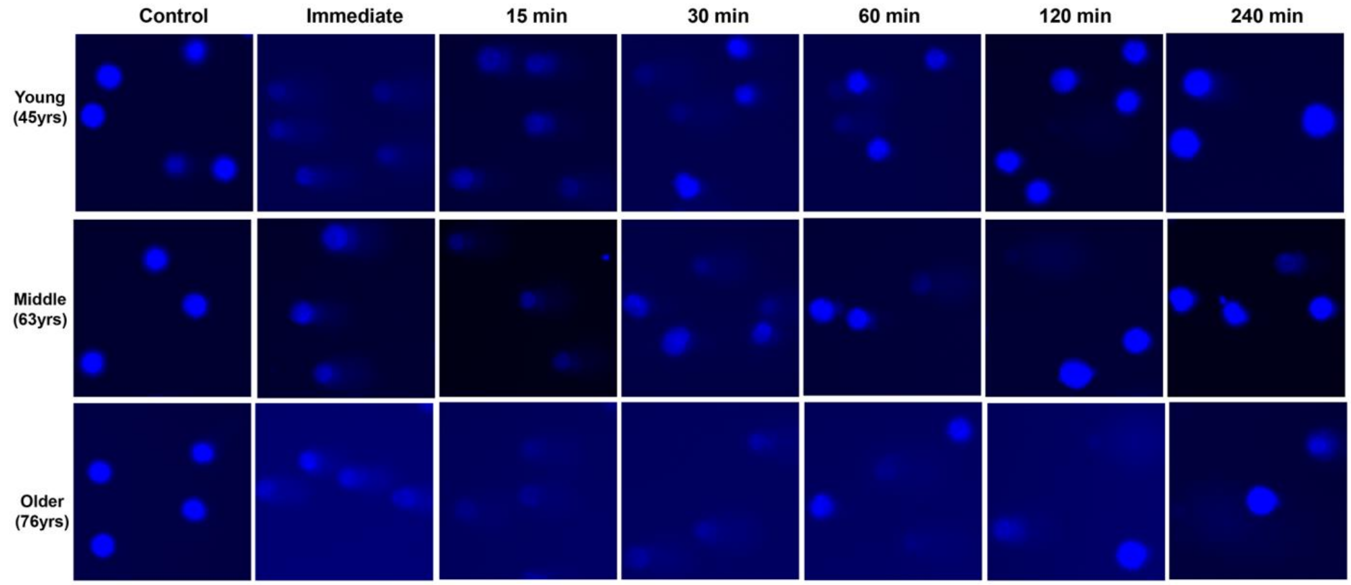

Supplementary Figure 1. Representative comet images during repair. Chondrocytes from young (45 years old), middle-aged (63 years old), and older (76 years old) donors were irradiated with 10 Gy and then imaged after various times of repair using the comet assay. The pictures shown represent the same donors for which individual cell data are plotted in Figure 2A.

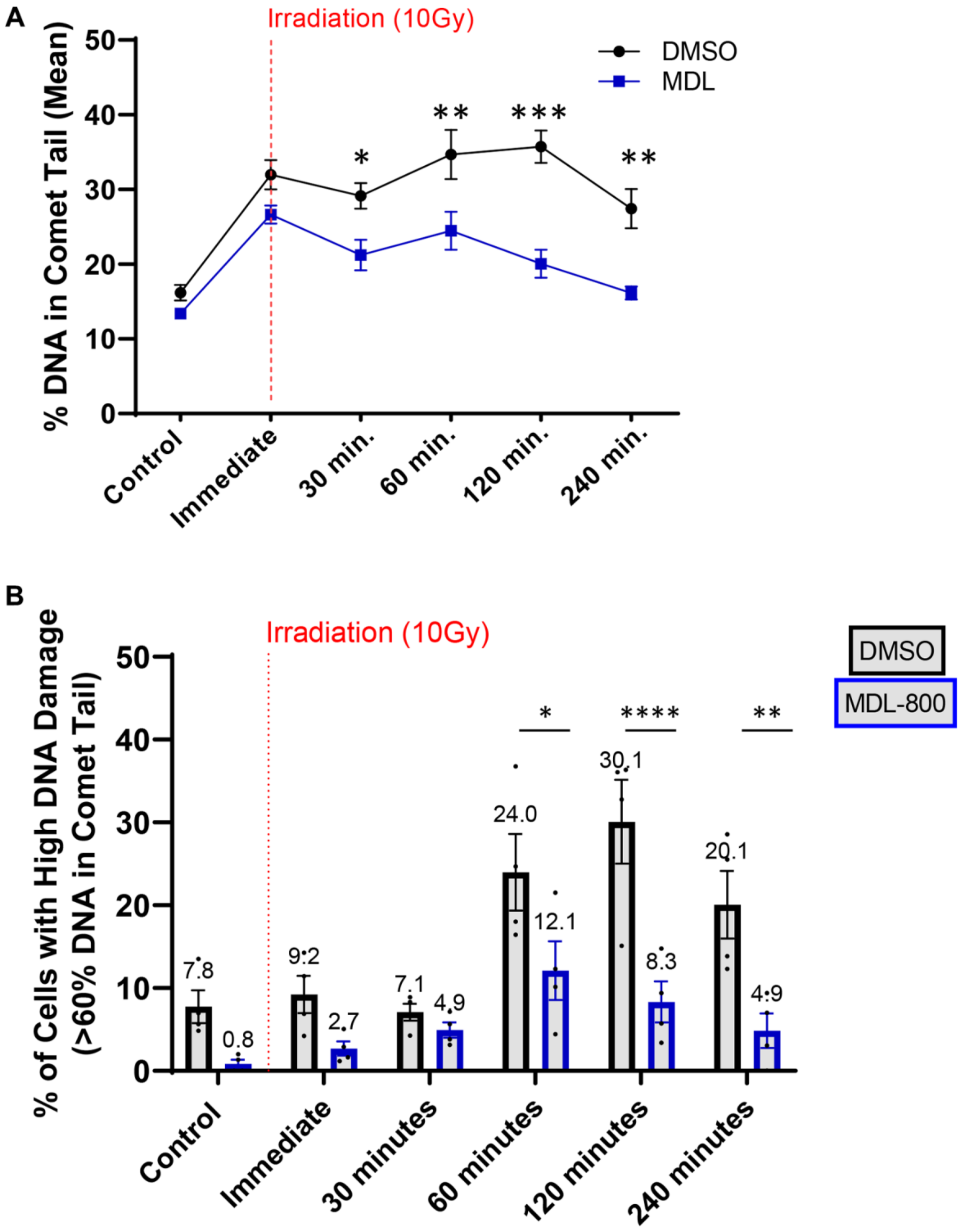

Supplementary Figure 2. Effect of SIRT6 activation and inhibition on older chondrocyte repair efficiency. Chondrocytes from older donors (*n* = 4, >70 years) were pre-treated with 20 µM MDL-800 or vehicle (DMSO) for 2 hours before trypsinization, gel encapsulation, and irradiation. Treatment continued during the repair phase. (**A**) The percentage of DNA in comet tails for all cells were averaged for each condition, and the mean of all donors per age group is shown (mean + SEM). Repair time, treatment, and their interaction were significant sources of variation (2-way repeated measures ANOVA). Significant differences between groups at each time point (Tukey's multiple comparisons test, $p < 0.05$) are denoted by symbols: (*) = DMSO vs. MDL. (**B**) The percentage of cells with high levels of DNA damage (>60% of DNA in comet tails). Statistics as in A.

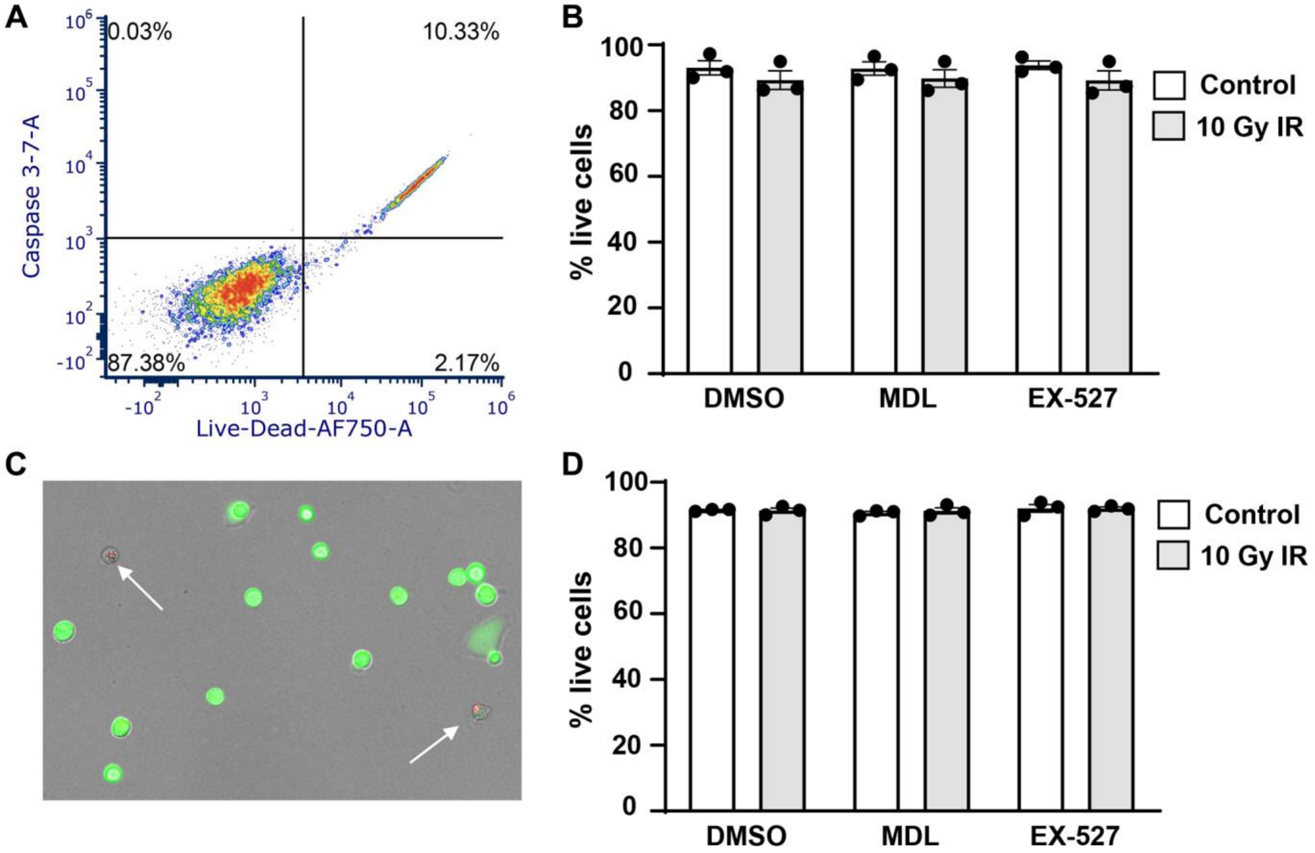

Supplementary Figure 3. Effect of SIRT6 modulation on apoptosis after irradiation. (**A**) Flow cytometry analysis after treatment with 20 µM MDL-800, 10 µM EX-527, or vehicle (DMSO) for 2 hours before and four hours after 10 Gy IR. (**B**) Quantification of the number of live/non-apoptotic chondrocytes (lower left quadrant) from three donors. (**C**) Representative image showing live cells (green) embedded within a low-melt agarose gel four hours after irradiation. White arrows indicate dead cells (lack of green, red stain for ethidium homodimer). (**D**) Quantification across chondrocytes from three donors.
